# Dentistry Amidst the COVID-19 Pandemic: Knowledge, Attitude, and Practices Among the Saudi Arabian Dental Students

**DOI:** 10.3389/fmed.2021.654524

**Published:** 2021-04-07

**Authors:** Basim Almulhim, Abdullah Alassaf, Sara Alghamdi, Riyadh Alroomy, Sami Aldhuwayhi, Abdullah Aljabr, Sreekanth Kumar Mallineni

**Affiliations:** ^1^Department of Preventive Dental Science, College of Dentistry, Majmaah University, Al-Majmaah, Saudi Arabia; ^2^Department of Restorative Dental Sciences, College of Dentistry, Majmaah University, Al-Majmaah, Saudi Arabia; ^3^Department of Prosthodontics, College of Dentistry, Majmaah University, Al-Majmaah, Saudi Arabia; ^4^Department of Medical Education, College of Dentistry, Majmaah University, Al-Majmaah, Saudi Arabia

**Keywords:** coronavirus, dentistry, dental students, practice, Saudi Arabia

## Abstract

**Background:** The recent pandemic outbreak has created a huge impact on dentistry. Dental students and dental professionals are at a higher risk because dental practice comprises close communication and widespread exposure to blood, saliva, and other body fluids. It is imperative to evaluate the knowledge and perceptions regarding Coronavirus (COVID-19) among budding dentists.

**Aim:** To assess the knowledge, attitude, and practices of dental students regarding dental practices during COVID-19.

**Materials and methods:** A cross-sectional questionnaire-based study was conducted among undergraduate students in Riyadh, Saudi Arabia. An online questionnaire consisting of demographic, knowledge, and attitude-based questions were circulated among the study population, and the responses for the knowledge and attitude were scored. Their mean scores were then calculated. Chi-square test and nonparametric tests were computed using SPSS version 21 software, and *p*-values < 0.05 were considered statistically significant.

**Results:** 388 undergraduate dental students have participated in the study from Saudi Arabia. 68% of the respondents believed that they had sufficient knowledge regarding COVID-19. The mean score for knowledge was 5.84 out of 7. Females (6.24) scored statistically significantly higher than males (5.55, *p* < = 0.001). The mean attitude score was 6.34 out of 9. 93% were using PPE models, while 95% maintained social distancing. Out of all the participating dental students, only 16% were willing to treat patients during the pandemic, and 28% did not want to treat patients, 28% preferred teledentistry. The majority (44%) of dental undergraduates were willing to handle only emergency cases.

**Conclusion:** Accurate knowledge and attitude regarding COVID-19 and diversified opinion on preventive practices during the pandemic period among budding dental professionals evident from Saudi Arabia. Mixed opinions were witnessed among them in seeking help from professional societies. The majority of dental undergraduates were willing to handle only emergency cases.

## Introduction

Shutters of dental offices were pulled down with dentistry flagged as a high-risk profession on the unforeseen advent of the highly contagious viral infection of severe acute respiratory syndrome coronavirus 2 (SARS-CoV-2), also known as COVID-19, in early 2020. The pandemic was declared by the World Health Organization (WHO) on Mar 11, 2020, following which, overnight, curfews and lockdowns had been implemented worldwide (1). It was identified that this highly infective virus is transmitted through direct and indirect modes. The natural mode of transmission took place via aerosols generated during surgical and dental procedures, in the form of respiratory droplet nuclei or other bodily secretions and fluids or mother-to-child. This increased the chances of a person getting infected if present in the proximity of 1m of the host. Indirectly, the transmission occurred on touching surfaces in the infected people's immediate environment or objects used by them (2).

The first dentist reported to have fallen prey to this virus was on January 23rd, 2020 the hospital at Wuhan University in China, a country where the infection is said to be originated from, and eventually other health professionals were tested COVID posted (3, 4). In context with this, the civic authorities urged delay of dental sittings unless there being an emergency, and thus, dentistry as a profession came to a standstill (5). The dental profession demands the patients violate the recommended one-meter safe distance and dental procedures generating aerosols. it was no surprise that all non-essential dental procedures were suspended as a part of the interim guidance (6). The oral health providers may accidentally provide direct care for infected or suspected yet not diagnosed COVID-19 patients. There have also been cases of asymptomatic infections wherein transmission may occur even before symptoms appeared (7, 8).

The pandemic took a toll on dental practices. A recent study by Wang et al. (9) reported that 138 hospitals admitted COVID positive patients in Wuhan and identified 29% of these hospitalized patients as healthcare workers. In contrast, Meng et al. reported nine COVID positive cases among dentists and students at Wuhan University, raising the alarm regarding healthcare professionals' safety, especially dentists (5). As much as 38% reduction in patients seeking dental treatment was observed by Guo et al. (10) during the Wuhan outbreak. Consequently, the authorities across the globe were jolted into formulating an action plan for dental services to be carried out post the initial interim suspension.

Several protocols have been set up to ensure minimum human-to-human contact during the pandemic. There is an overall consensus of screening patients prior to initiation of any dental procedures and triage. The dentists are advised to don personal protective equipment (PPE) kits, facemasks, preferably N-95, and face shields. Minimization of aerosol generation, adequately ventilated operatory, and strict compliance with infection control measures and bio-waste management are reinforced (11). Practices of “teledentistry” or web/ telephonic consultation are also being encouraged (12). The face of dentistry is changing, with COVID-19 presenting an unprecedented challenge to the dental industry. The future and sustenance of practicing and emerging dental professionals depend on their adherence to these new norms and protocols and adapting to post-COVID dentistry 2.0. Dentistry has been forced to evolve right from how dental education is imparted to how dental procedures are carried out. Upon identifying the scarcity of such studies conducted to date, this study aims at evaluating the knowledge, attitudes, and practices of undergraduate students in Saudi Arabia to assist in preparing the dental workforce in responding better to such pandemics.

## Methods

This cross-sectional questionnaire-based study has been carried out following the STROBE guidelines (13) specified for this type of study upon approval was obtained from the Institution Ethical Committee, Majmaah University under IRB No. MUREC-June,03/COM-202-/31-2. The study was conducted in Saudi Arabia among undergraduate dental students to investigate their knowledge, attitude, and preventive practices toward COVID-19 from 01-06-2020 to 25-09-2020. Only dental undergraduate students and included both genders. Only those who were willing to sign a written informed consent participated in the study. Postgraduates, Faculty, Private practitioners, those working outside Saudi Arabia, and other health care professionals were excluded from the study. The sample size was calculated using the Raosoft online sample size calculator (14). Based on the survey by Althomairy et al. (15) in an assumption of 3,000 active Saudi dental society members, a response distribution of 50%, while the margin of error and confidence intervals of 5 and 95%, respectively, were made to reach a sample size of 341 dental students (15).

A structured online self-administered questionnaire was administered for this cross-sectional survey to evaluate knowledge, attitudes, and practices regarding COVID-19 among dental undergraduate students. The questionnaire was divided into five main parts. The first constituted the study participants' demographic information (age and gender). The second part explored (i) Information about Coronavirus in your professional society sufficient, (ii) Government institutions able to control the pandemic, and (iii) Obtaining sufficient knowledge regarding COVID-19 information. The third part elicited knowledge of oral health professionals toward COVID-19 by giving correct or incorrect options for every question. The fourth part assessed attitudes toward COVID-19 infection. The fifth part was considered for preventive practice. Each response was scored as “1” (correct) and “0” (wrong), with knowledge scores ranging from 0 to 7 and attitude scores ranging from 0 to 9. Comparisons of knowledge and attitude scores based on gender and practice questions were evaluated. The correlation of knowledge and attitude scores was established based on practice questions. The questionnaire was circulated among the target population in the form of an online Google form, and the recruitment was done through various social media platforms and contact information obtained from the database. The data assimilated was analyzed using Statistical Package for Social Sciences (SPSS) version 21.0 software NY, USA (16). One–way-ANOVA with Bonferroni corrections was used to analyze more than three groups. A Chi-square test was computed to determine the association between study variables. All the tests were assessed at a 5% level of significance.

## Results

**Figure 1:**
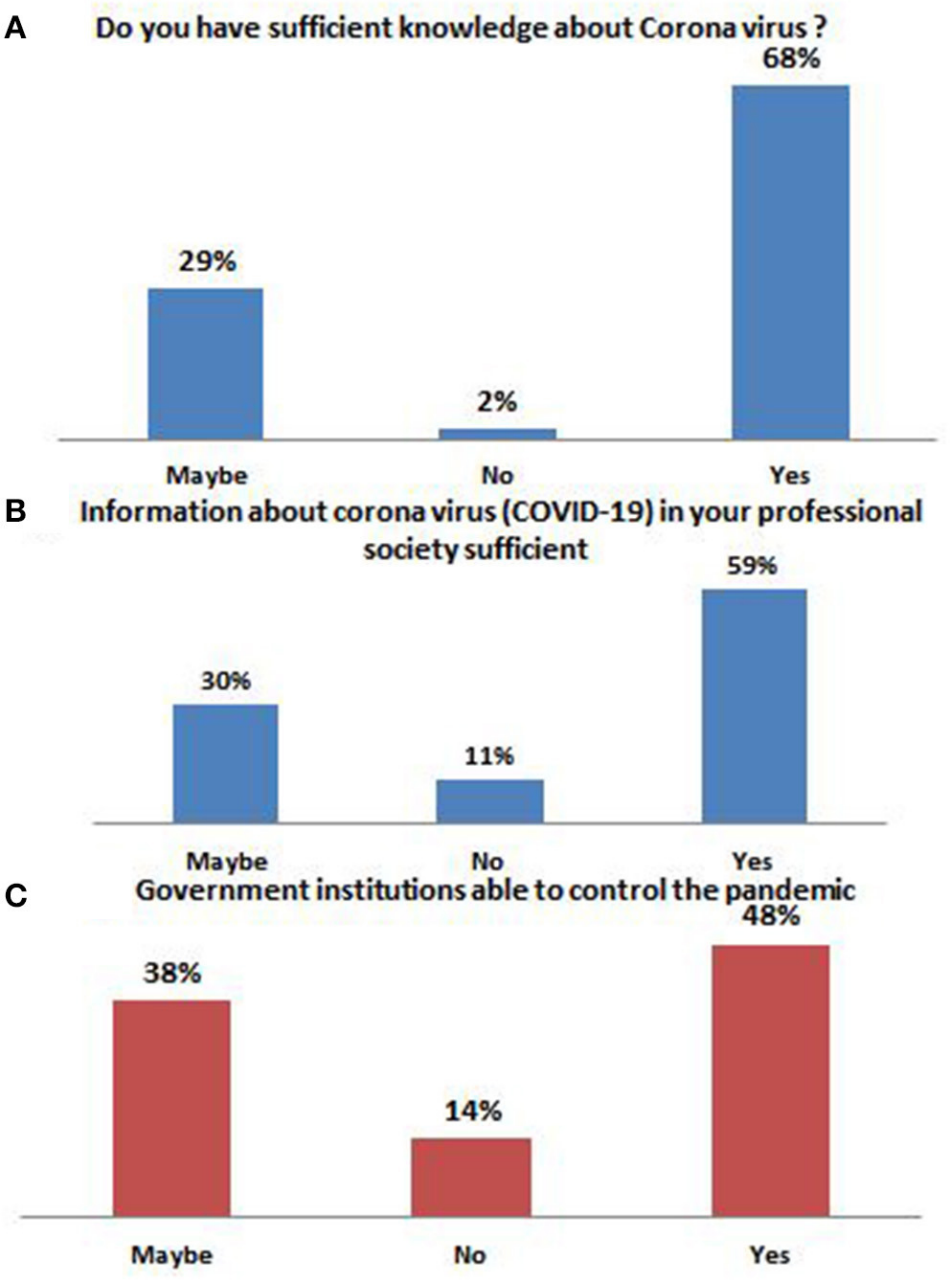
Arabian undergraduate dental students opinions **(A)** sufficient knowledge about Corona virus (COVID-19) **(B)** available information about COVID-19 in your professional society sufficient and **(C)** the role government societies during COVID-19.

A total of 388 responses were received from undergraduate dental students, out of which 223 respondents were male while the remaining 165 were females with a mean age of 22 years. Amongst the participants, Saudis were 91%, and non-Saudis were 9%. When questioned about the knowledge regarding COVID-19, most of the participants (68%) believed that they had sufficient knowledge, while 29% were unsure, only 2% felt they didn't have the required knowledge (Figure 1). 48% thought that adequate information was available in their professional society, 38% were unsure, and 14% found the available information insufficient. Government institutions' ability to control the on-going pandemic was found satisfactory by the majority (59%) of the students, but 30% and 11% were unsure and found it insufficient, respectively (Figure 1). The mean score for knowledge was observed to be 5.84, where the maximum score was 7 (Table 1). Females (6.24) scored statistically significantly higher than males (5.55, *p* < 0.001). The mean attitude score was 6.34, where the maximum score was 9, and here too, females (6.53) managed to secure a higher mean score than males (6.19) (Table 2). Surprisingly, only 16% were treating patients during the pandemic, and 28% did not want to treat patients; 28% preferred teledentistry while a majority of 44% were willing to handle only emergency cases (Table 3). A 93% of the dental students revealed the use of PPE models when asked questions concerning prevailing practices and following protocols. They also had better knowledge, and attitude mean scores (5.88, 6.38) than those who didn't use PPE models (5.34, 5.72), summarized in Table 4. Social distancing was maintained by 95% of the undergraduates who had also scored statistically significantly higher than their peers on the attitude questions (6.44 > 4.3, *p* < 0.001) (Table 5). The 16% treating patient had statistically significantly higher mean attitude scores than those who did not treat patients (7.31 > 6.15, *p* < 0.001) but had lower knowledge mean scores (5.65 < 5.87, *p* = 0.225) (Table 6). No statistically significant difference was found in the mean knowledge and attitude scores when comparisons were made between those undergraduate dental students who preferred to teledentistry, who did not want to treat patients during the lockdown, and who only treated emergency patients using Bonferroni comparison (Table 7).

**Table 1:** Overall knowledge and attitude scores on COVID-19 among Arabian undergraduate dental students.

**Overall total score**	**Mean**	**Standard deviation**	**Median**	**Mode**	**Percentile 25**	**Percentile 75**
Knowledge (max = 7)	5.84	1.31	6.00	7.00	5.00	7.00
Attitude score (max = 9)	6.34	1.83	7.00	7.00	5.00	8.00

**Table 2:** Comparison of knowledge and attitude scores on COVID-19 among Arabian undergraduate students based on gender.

**Details**	**Female (*****n*** **= 165)**		**Male (*****n*** **= 223)**		**Mean Difference 95% Confidence Interval**	***p*-value**
	**Mean**	**Standard deviation**	**Mean**	**Standard deviation**		
Knowledge	6.24	0.83	5.54	1.50	0.69 (0.44 to 0.95)	0.00^*^
Attitude	6.52	1.47	6.19	2.038	0.33 (−0.03 to 0.70)	0.07

* *p < 0.05 is considered statistically significant*.

**Table 3:** Responses of undergraduate students on practice-based questions during COVID-19.

**Question**	**Response**	***N* (%)**
**Are you using the personal protection equipment for safety?**	No	29 (7)
	Yes	359 (93)
**Are you maintaining social distancing?**	No	20 (5)
	Yes	368 (95)
**Are you treating your patients now?**	No	327 (84)
	Yes	61 (16)
**Do you want to treat patients during this lockdown?**	I prefer online (tele dentistry) consultation	109 (28)
	No	108 (28)
	Only Emergency patients	171 (44)

**Table 4:** Comparison of knowledge and attitudes scores based on use of personal protection equipment.

**Using personal protection equipment**	**No (*****n*** **= 29)**		**Yes (*****n*** **= 359)**		**Mean Difference 95% Confidence Interval**	***p*-value**
	**Mean**	**Standard deviation**	**Mean**	**Standard deviation**		
Knowledge	5.34	1.31	5.88	1.30	−0.53 (−1.03 to −0.04)	0.03^*^
Attitude	5.72	1.19	6.38	1.86	−0.66 (−1.3 to 0.03)	0.06

* *p < 0.05 is considered statistically significant*.

**Table 5:** Comparison of knowledge and attitudes scores based on social distancing.

**Social distancing**	**No (*****n*** **= 20)**		**Yes (*****n*** **= 368)**		**Mean Difference 95% Confidence Interval**	***p*-value**
	**Mean**	**Standard deviation**	**Mean**	**Standard deviation**		
Knowledge	5.8	0.89	5.84	1.32	−0.04 (−0.63 to 0.54)	0.88
Attitude	4.3	1.62	6.44	1.77	−2.14 (−2.94 to −1.34)	0.00^*^

* *p < 0.05 is considered statistically significant*.

**Table 6:** Comparison of knowledge and attitudes scores based on their practice in the dental operatory.

**Treating patients**	**No (*****n*** **= 327)**		**Yes (*****n*** **= 61)**		**Mean Difference 95% Confidence Interval**	***p*-value**
	**Mean**	**Standard Deviation**	**Mean**	**Standard Deviation**		
Knowledge	5.87	1.28	5.65	1.43	0.22 (−0.130.58)	0.22
Attitude	6.15	1.83	7.31	1.42	−1.15 (−1.64 to −0.67)	0.00^*^

* *p < 0.05 is considered statistically significant*.

**Table 7:** Comparison of practice-based responses of Arabian undergraduate dental students.

**Dependent Variable**	**Do you want treat patients during this lock down?**		**Mean Difference (95% Confidence Interval)**	***P*-value**
			
Knowledge	I prefer online (tele dentistry) consultation	No	−0.01(−0.43 to 0.42)	1.000
		Only Emergency patients	−0.054 (−0.44 to 0.33)	1.000
	No	Only Emergency patients	−0.05 (−0.43 to 0.34)	1.000
Attitude	I prefer online (tele dentistry) consultation	No	0.22 (−0.37 to 0.81)	1.000
		Only Emergency patients	−0.15 (−0.68 to 0.38)	1.000
	No	Only Emergency patients	−0.36 (−0.90 to 0.17)	0.304

*p < 0.05 is considered statistically significant.

## Discussion

During the period of complete pandemonium across the globe, Saudi Arabia announced its first COVID-19 positive case on 2nd March 2020, and by 7th June, 2020, the total for confirmed cases had surpassed 1,00,000(17, 18). This unexpected outbreak put a strain on health systems of several countries and demanded that the health professionals step-up and accept the role of “Corona Warriors.” Amidst the waves of rapidly transmitting infections was a wave of “mis-infodemic,” which spread incorrect information, resulting in panic among the general population. The governments implemented varied guidelines, restrictions, bans, and curfews worldwide based on their healthcare systems, economies, and political ideologies. COVID-19 epidemics, etiology, clinical findings, epidemics, and treatment options necessitate widespread data from clinical trials (6, 19, 20). It has also been suggested that rapid discoveries can control the spread and further outbreaks of COVID-19. This disease spreads person-to-person, either through direct transmission by cough, sneeze, or droplet inhalation, or mucous membranes, or ocular contact of the eyes and saliva (8, 9, 19, 21). As a result of this hullabaloo, dental professionals had to face several challenges in the form of initial suspension of practice and later safe treatment of patients abiding by the prescribed infection control protocols (19). This study makes an effort to understand undergraduate dental student's knowledge, attitudes, and practices- training about COVID-19 and the transformed post- pandemic dentistry.

The current study evaluated the participating dental undergraduate students' knowledge by scoring them based on seven knowledge-based questions. The study demonstrated adequate knowledge about COVID-19 with a mean score of approximately six. This was in agreement with a previous study conducted by Srivastava et al. among dental health care professionals (22). Similarly, in a survey by Quadri et al. (23), dental interns, auxiliaries, and specialists scored above average scores when their knowledge was tested. However, in contrast to these findings, a prior study conducted in Georgia among the dental health care workers reported that more than half of the dental students, residents, and specialists did not have enough knowledge of viral infections (24). No prior studies have identified the correlation between gender and knowledge about COVID-19, whereas the current study reports a statistically significant difference in males' and females' knowledge, with females scoring better (*p* ≤ 0.001).

In situations of public health crises like this, one's belief in government institutions' efforts shapes their morale, feeling of safety while also motivating them to effectively carry out duties assigned to them and ultimately be reflective in the nation's fight against the pandemic. 59% of the current study participants found government institutions' efforts satisfactory, others being unsure or finding it lacking. A similar study by Rabbani et al. (25) had found 75% of the Saudi Arabian healthcare workers unsatisfied with their institution's preparedness for the pandemic. These professionals' attitudes are associated with their practices, as noticed in this study wherein those with higher mean attitude scores were observed to practice social distancing. This difference was found to be statistically significant (*p* ≤ 0.001).

After the interim suspension of non-essential dental treatments, new protocols have been imposed to resumption safe treatment of all cases. 84% of the participants have started treating patients. The respondents were questions about their adherence to safety protocols, and it was noted that 93% wore PPE models and 95% maintained social distancing guidelines. These numbers indicate awareness of required measures and implementation of set standards for infection control (26). However, despite relaxation regarding dental procedures that are allowed and precautionary measures, the majority (44%) still preferred to handle only emergency cases.

In the present study, accurate scores were obtained for knowledge and attitude by undergraduate students regarding COVID-19. Diverse scores were evident regarding preventive practice during COVID-19.especilly for the question on dental practice. Similarly, a recent Arabian study (27) found adequate knowledge and attitude scores and low practice scores, and the authors conducted the study with postgraduate and undergraduate students. Hence, the findings were not compared with the present study. COVID-19 plays a vital role in its control and prevention and would help formulate appropriate protocols and reform the prevailing practices to combat such pandemics in the future. The knowledge and attitudes scores of Arabian dental undergraduates were evaluated based on practices. Amongst these, overall knowledge mean scores showed correlation with the use of PPE, while social distancing and treating patients during pandemic showed correlation with attitude mean scores. Almost similar scores on knowledge among dental healthcare professionals regarding COVID-19 were reported (28, 29). The present study was conducted with dental undergraduate students; However, these findings with previous studies were not comparable. An interesting finding was the preference of teledentistry by 28% of the dental students. Teledentistry is an innovative way of rising against the challenge of social distancing by utilizing available information and technologies using smartphones, tablets, and computers for live video or phone consultation recorded videos, etc. (11, 30). It comprises remote assessment of patients with teleconsultation or telediagnosis, triage of patients, telemonitoring, and dental care provision remotely when possible and appropriate to the patients (9, 11, 31, 32). This pandemic has left many challenges to the dental profession, and it became mandatory to evaluate the knowledge and perception of oral healthcare professionals (9, 21). Prior surveys have shown this form of remote consultation gaining popularity for treatment for disabled, elderly, and patients who do not have access to health services and during the COVID-19 crises (31–33). COVID-19 plays a vital role in its control and prevention and would help formulate appropriate protocols and reform the prevailing practices to combat such pandemics in the future.

According to the best of our knowledge, this study is first conducted among undergraduate dental students in the Riyadh region, Saudi Arabia. However, while interpreting this study's results, certain limitations that this study had encountered need to be kept in mind. Firstly, due to the strict lockdowns imposed, face-to-face interviews were infeasible, resulting in this survey being conducted online, indicating the respondents' not having understood the questions accurately. The data assimilated has been self-reported by the respondents; thus reporting bias cannot be completely ruled out. In the present study dental undergraduate from Saudi Arabia was involved, and the authors tried to establish their perception of practicing dentistry during the pandemic period. It is a fact that dental undergraduate cannot do their own dental practices. Another limitation was using self-reported measures, convenience sampling, and a cross-sectional design, which cannot be generalized to the study findings. College and their province in Saudi Arabia of the dental undergraduate students did not take for evaluation. This might also make a difference that could be considered a potential limitation of the study. The questionnaire was sent to participants via social media, and we received only 388 responses; hence, the response rate was not sought. The present study looked into overall mean scores. The individual scores were not taken into consideration for the analysis. This also could be a potential limitation of the present study. Even though the dental undergraduates were not allowed to practice independently, the present study determines their knowledge and attitudes, and practices in the Arabian region. This study opens the arena to carry out nation-wide studies targeting a larger population from various regions of Saudi Arabia as well as in other counties to have a global comparison. Healthcare professionals form the backbone of any country's public health, and dentists play a vital role in this, with dental health being an integral part of general health. Correct knowledge about any disease plays an essential role in its control and prevention, especially in cases where the population is susceptible.

## Conclusions

Accurate knowledge and attitude regarding COVID-19 and diversified opinions on dental practice during pandemic were evident among budding dental professionals from Saudi Arabia. The study observed mixed opinions among them in seeking help from professional societies. The majority of dental undergraduates were willing to handle only emergency cases.

## Data Availability Statement

The raw data supporting the conclusions of this article will be made available by the authors, without undue reservation.

## Ethics Statement

The studies involving human participants were reviewed and approved by Majmaah University, Saudi Arabia. Written informed consent for participation was not required for this study in accordance with the national legislation and the institutional requirements.

## Author Contributions

BA: study conception. AAla and SAlg: study design. AAlj, BA, and SM: data collection. RA, SAlg, and SAld: data analysis and manuscript drafting. AAla and SM: data interpretation. RA, SAld and SM: critical revision of the manuscript. All authors approval of the final version. All authors contributed to the article and approved the submitted version.

## Conflict of Interest

The authors declare that the research was conducted in the absence of any commercial or financial relationships that could be construed as a potential conflict of interest.
