# Supplementary material for: Dentistry Amidst the COVID-19 Pandemic: Knowledge, Attitude, and Practices Among the Saudi Arabian Dental Students
Source: Front Med (Lausanne). 2021 Apr 7;8:654524. doi: 10.3389/fmed.2021.654524 (PMC8058223; doi:10.3389/fmed.2021.654524)
Supplement: Supplementary file 1 [file Data_Sheet_1.PDF]

Questionnaire form

Gender (Male/ Female)
Are you? (Saudi / Non-Saudi)
Age
Do you have sufficient knowledge about Corona virus (COVID-19)?
Is the available information about corona virus (COVID-19) in your professional society sufficient?
Are the government institutions able to control the pandemic?
**Knowledge**
Do you know that Coronavirus (COVID-19) is a viral infection?
Do you know that the Corona virus (COVID-19) is fatal?
Do you know that the Corona virus (COVID-19) is transmitted by close contact with an infected person?
Do you know that fever, cough, and shortness of breath are symptoms of the Corona virus (COVID-19)?
Do you know that the use of a mouth mask can help in avoiding Coronavirus (COVID-19)?
Do you know dentists are at a higher risk of Coronavirus (COVID-19)?
Do you know that the incubation period is Coronavirus (COVID-19) is 2 weeks?
**Attitudes**
Do you think the vaccine is available in markets?
Do you think washing hands with soap and water or sanitizer can help prevent Coronavirus (COVID-19) transmission?
Do you think patients with underlying chronic diseases are at a higher risk of Coronavirus (COVID-19)?
Are you worried one of your family members may get Coronavirus (COVID-19)?
Transmission of Coronavirus (COVID-19) can be prevented by using standard and isolation precautions given by WHO, CDC, ADA, etc?,
Prevalence of Coronavirus (COVID-19) can be reduced by the active participation of health care workers in hospital infection control programs?
If a Coronavirus (COVID-19) vaccine is available, would you have it?
Do you think intensive treatment should be given to diagnosed patients with Coronavirus (COVID-19) positive?
Health care workers must avail themselves of all information about the Coronavirus (C-19)?
**Practice**
Are you using personal protective equipment for safety?
Are you maintaining social distancing?
Are you treating your patients, now?
Do you want treat patients during this lock down?
